# Preliminary Study on Patient-Reported Pain and Early Functional Outcomes of Robotic Arm-Assisted Versus Jig-Based Total Knee Arthroplasty

**DOI:** 10.7759/cureus.80812

**Published:** 2025-03-19

**Authors:** Sangeeta S Babu, Kalesh Kavumpurath, Salil Mohammed, Druvan Shaji, Jai Thilak

**Affiliations:** 1 Physical Medicine and Rehabilitation, Amrita Institute of Medical Sciences and Research Center, Kochi, IND; 2 Orthopaedics, Amrita Institute of Medical Sciences and Research Center, Kochi, IND; 3 Orthopaedics and Traumatology, Amrita Institute of Medical Sciences and Research Center, Kochi, IND

**Keywords:** arthroplasty, knee, osteoarthritis, pain, physiotherapy, robotics

## Abstract

Purpose: Robotic knee replacement has gained widespread popularity globally, although the functional outcomes for patients, in comparison to traditional surgery, remain uncertain. This study aimed to compare patient-reported pain levels and early functional outcomes following robotic arm-assisted total knee arthroplasty (RTKA) and conventional jig-based TKA. The focus was on evaluating differences in pain relief, functional recovery, and postoperative scores at three, six, and 12 months.

Methods: A retrospective analysis was conducted on 240 patients with tri-compartmental osteoarthritis who underwent primary TKA between January 2021 and September 2022. Of these, 120 received RTKA, and 120 underwent conventional TKA. Patients were assessed preoperatively and postoperatively at three, six, and 12 months using the Western Ontario McMaster University Osteoarthritis Index (WOMAC), Hospital for Special Surgery (HSS) Knee Rating Scale, and Oxford Knee Score (OKS). Statistical analyses, including t-tests, Mann-Whitney U tests, and Friedman analysis of variance (ANOVA), were performed to compare differences in outcomes.

Results: Both the RTKA and conventional TKA groups exhibited significant functional improvements from baseline across all evaluated measures. When comparing absolute postoperative scores, there were no significant differences in WOMAC and OKS scores between the groups at three, six, and 12 months (p-values: 0.198, 0.206, and 0.446 for WOMAC; 0.465, 0.117, and 1.0 for OKS). However, the RTKA group had significantly higher HSS scores at 3 months (p = 0.032) and showed a significantly greater improvement in HSS from baseline at three months (p = 0.004) and in OKS from baseline at six months (p = 0.037). By 12 months, no notable differences in functional outcomes were observed between the groups. Patient satisfaction was high in both groups, with a trend toward greater satisfaction in the RTKA group regarding pain relief and daily activities.

Conclusions: RTKA provided significant improvements in early functional outcomes, as evidenced by the higher HSS score at three months and the greater improvement in OKS at six months compared to baseline. However, by 12 months, no significant differences were observed between RTKA and conventional jig-based TKA in terms of functional outcomes and pain relief. These findings suggest that while robotic technology may enhance early recovery, its long-term benefits remain uncertain. Further research with extended follow-up periods especially in personalized alignment philosophies is necessary to evaluate the potential advantages of robotic assistance in long-term functional outcomes and implant longevity.

## Introduction

Knee osteoarthritis is a degenerative joint condition that leads to pain, stiffness, and deformity, restricting the range of motion as the condition worsens [1]. Over the past two decades, there has been a steady increase in the global incidence of total knee arthroplasty (TKA) [2]. To address the challenges posed by knee osteoarthritis, TKA has become a common and effective procedure [3]. Robotic arm-assisted TKA (RTKA) has gained significant attention for its potential to improve patient satisfaction and long-term outcomes through enhanced accuracy and precision in prosthetic component placement [4].

The alignment of the limb and the accuracy of implant positioning are crucial factors that influence the long-term durability of the implant, clinical results, functional performance, and patient satisfaction [5]. Robotic knee arthroplasty has been shown to improve the precision of implant placement, which, in turn, enhances clinical and functional outcomes following the procedure [6].

The RTKA system (Mako, Stryker, Mahwah, New Jersey, USA) is a semi-active system that enables interaction between the surgeon and the robotic machine during implant alignment, bone preparation, and knee balancing. These surgeon-controlled factors affect long-term survival, implant stability, and patient outcomes [7]. Postoperative physical therapy is also vital for restoring muscle strength and range of motion after knee replacement [8]. While robotic assistance has improved implant alignment, its effects on clinical scores, patient satisfaction, and implant longevity are still being studied [9].

Although knee replacement surgery is generally successful, only 70-86% of patients report being satisfied with the outcome [10]. Compared to traditional TKA, RTKA does not show significantly higher short- and mid-term knee outcome scores [11]. However, RTKA has led to more accurate component placement, reduced alignment errors, and better patient-reported outcomes than manual jig-based procedures [12]. Many studies have shown that, after RTKA, patients experience greater satisfaction, shorter recovery times for independent movement, and improved knee function during daily activities [13].

Despite the benefits, knee balancing in traditional TKA is primarily based on the surgeon's subjective assessment, which can vary across procedures [14]. RTKA has demonstrated improved alignment of the knee prosthesis, but its impact on functional outcomes remains uncertain [4]. While robotic surgery has enhanced the precision of component placement and minimized errors, both short-term and long-term outcomes have not shown significant improvements in implant survival, pain levels, or functional performance. Although many studies have focused on implant positioning in CT-guided robot-assisted TKA (RA-TKA), few have directly compared the functional outcomes of conventional and RTKA [15]. This retrospective study aims to address this gap in research.

## Materials and methods

This retrospective study included 240 patients with painful tri-compartmental osteoarthritis who underwent primary TKA between January 2021 and September 2022. Of these participants, 120 received RTKA, while the other 120 underwent conventional jig-based TKA, with no randomization in the allocation. Approval was obtained from the institutional ethics committee before the study began, and informed consent was collected from all participants. The sample size calculation, based on the average Western Ontario McMaster University Osteoarthritis Index (WOMAC) score, determined that a minimum of 120 individuals per group (240 total) was required.

Statistical analyses were performed using Statistical Package for the Social Sciences (IBM SPSS Statistics for Windows, IBM Corp., Version 20.0, Armonk, NY). Categorical variables were presented as frequencies and percentages, while continuous variables were expressed as either the mean (with standard deviation) or median (with interquartile range, Q1-Q3). The independent sample t-test was used to assess the statistical significance of the mean age differences between the two groups, and the Mann-Whitney U test and t-test were used to evaluate median score differences. The Friedman two-way analysis of variance (ANOVA) and the Mann-Whitney U test were used to assess the statistical significance of median changes in various scores from baseline to post-surgery.

Patient selection was based on clinical assessments, medical history, and radiological analysis, following specific inclusion and exclusion criteria. The inclusion criteria were as follows: patients with primary knee osteoarthritis, a varus deformity of less than 20°, a valgus deformity of less than 10°, aged between 55 and 80 years, skeletally mature, and receiving primary TKA for degenerative arthritis, regardless of gender. Patients with a history of previous knee surgery, post-traumatic, including those for septic arthritis, high tibial osteotomy, meniscal repairs, a body mass index exceeding 40 kg/m^2^, inflammatory arthritis, and skeletal immaturity were excluded from the study.

Baseline demographic data showed no significant differences between the two groups - those receiving RTKA and those undergoing conventional TKA. All surgeries were performed by a single senior surgeon with 20 years of experience in conventional jig-based TKA and three years in RTKA. Both groups followed the same mechanical alignment principles. The Mako robotic arm is an image-based, semi-automatic system that uses a preoperative CT scan to generate a 3D image of the patient’s knee. In RTKA procedures, the Triathlon cruciate-retaining total knee system (Stryker) was utilized, with all patients receiving the Triathlon X3 patella replacement. In contrast, conventional TKA procedures used the Maxx Destiknee cruciate-sacrificing system (Meril Life Sciences, Vapi, India).

The post-operative rehabilitation protocol was uniformly followed across both groups, with all patients beginning ambulation on the day of surgery. Evaluations were conducted before surgery and at three, six, and 12 months postoperatively. All patients were included in the follow-up, and data were collected by a single observer. There was no significant difference in the baseline data between the patients who underwent traditional jig-based TKA and those who had RTKA.

The surgeries were performed using a medial parapatellar approach under spinal anesthesia, followed by an adductor canal block. A standardized postoperative protocol was implemented, starting after patients were transferred from the surgical recovery unit to the ward, approximately five hours post-surgery. Pain scores were recorded from the day of surgery until discharge. The rehabilitation program included ankle pumps, static quadriceps and hamstring exercises, active knee flexion, straight leg raises, short arch exercises, knee extension exercises, and full weight-bearing walking, all starting from the day of surgery. Physiotherapy sessions lasted at least 30 minutes each, and the same interdisciplinary team conducted rehabilitation for all patients in both groups.

Patients were instructed to use a compression bandage to reduce swelling and perform ankle pumps every two hours, excluding nighttime, with 10 repetitions per session and five sessions daily, to prevent deep vein thrombosis. All patients were discharged on the third postoperative day once they achieved adequate pain management, knee flexion of at least 90 to 100 degrees, and could walk independently with assistance from a walker, crutches, or a cane (supports were discontinued shortly after if the patient was confident in walking steadily). Patients were sent home rather than to a rehabilitation center or skilled nursing facility.

The first follow-up evaluation was scheduled for six weeks post-surgery. An unbiased observer recorded the preoperative and postoperative outcomes for each participant. Pain levels were assessed using a numerical rating scale ranging from 1 to 10, representing the spectrum from best to worst. Knee flexion was measured preoperatively and at discharge using a goniometer. The study also tracked the number of inpatient physiotherapy sessions, the time taken to achieve a straight leg lift in the supine position, and the length of hospital stays. Postoperative complications were monitored at three, six, and 12 months. Functional outcomes were assessed using the WOMAC, Hospital for Special Surgery Knee Rating Scale (HSS), and Oxford Knee Score (OKS), both preoperatively and at three, six, and 12 months post-surgery. Patient satisfaction was assessed 12 months after surgery using a 5-point Likert scale.

## Results

A total of 120 patients underwent RTKA, while another 120 patients received conventional TKA, with both groups having an average age of 65 years.

One year after the RTKA procedure, 116 out of 120 patients reported high satisfaction with pain relief, while four expressed moderate satisfaction. Regarding their ability to perform yard work, 117 patients were highly satisfied, and three reported moderate satisfaction. In terms of recreational activities, 111 patients were very satisfied, eight were slightly satisfied, and one was somewhat dissatisfied.

One year after conventional TKA, 109 of the 120 patients expressed high satisfaction with pain management, while 11 had moderate satisfaction. As for yard work, 110 patients were highly satisfied, and 10 were moderately satisfied. For recreational activities, 104 patients were highly satisfied, and 16 reported moderate satisfaction (Table 1).

**Table 1:** Comparison of satisfaction between two groups SS: somewhat satisfied; VS: very satisfied; SD: somewhat dissatisfied; TKA: total knee arthroplasty

	Response	Robotic TKA (n=120), n (%)	Conventional TKA (n=120), n (%)	p-values
Pain relief satisfaction	SS	4 (3.3)	11 (9.2)	0.107
	VS	116 (96.7)	109 (90.8)	0.107
Overall satisfaction	SS	7 (5.8)	3 (2.5)	0.333
	VS	113 (94.2)	117 (97.5)	0.333
Ability to perform home/yard work	SS	3 (2.5)	10 (8.3)	0.084
	VS	117 (97.5)	110 (91.7)	0.084
Ability to perform recreational activities	SD	1 (0.8)	0	-
	SS	8 (6.7)	16 (13.3)	-
	VS	111 (92.5)	104 (86.7)	-

The study results indicate that there were no statistically significant differences in the WOMAC scores at three, six, and 12 months after surgery between patients who underwent RTKA and those who received conventional TKA, with p-values of 0.198, 0.206, and 0.446, respectively. Similarly, no notable differences were found in the OKS at three, six, and 12 months postoperatively, with p-values of 0.465, 0.117, and 1, respectively.

**Figure 1:**
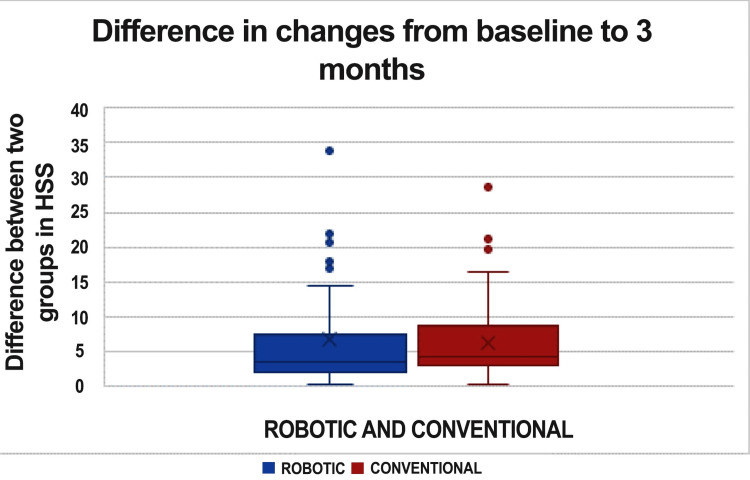
The graph represents changes in HSS score from baseline to three months HSS: Hospital for Special Surgery Knee Rating Scale

In the three-month postoperative phase, the HSS scores between the two groups exhibit a significant disparity (Figure 1).

Among the 120 patients in each group, 69 patients from the conventional TKA group demonstrated better functional outcomes than those in the RTKA group, while 48 patients from the RTKA group achieved superior functional outcomes compared to their counterparts in the conventional TKA group. Additionally, three patients in each group reported identical scores. After three months, there was a significant difference in HSS scores between patients who underwent RTKA and those who had conventional TKA, with a p-value of 0.032. However, no significant differences were observed between the two groups at six months (p-value = 0.969) or 12 months (p-value = 0.463) (Table 2).

**Table 2:** Comparison of satisfaction between two groups TKA: total knee arthroplasty; pre-op: pre-operative; ROM: range of motion; WOMAC: Western Ontario McMaster University Osteoarthritis Index; HSS: Hospital for Special Surgery Knee Rating Scale; OKS: Oxford Knee Score

Variables	Group		p-value
	Robotic TKA (n=120)	Conventional TKA (n=120)	
Mean age (years)	65.23 (6.68)	65.55 (8.12)	0.735
Sex, n (%)			
Female	103 (85.8)	94 (78.3)	0.130
Male	17 (14.2)	26 (21.7)	0.130
Pre-op ROM (degrees)	100 (90-110)	100 (80-100)	0.187
WOMAC			
Pre-op score	70.46 (44-81)	69.90 (50-83)	0.535
After 3 months score	9.68 (0.00-38)	8.46 (0.00-37)	0.198
After 6 months score	2.98 (0.00-25)	2.18 (0.00-34)	0.206
After 12 months score	0.22 (0.00-9.00)	0.33 (0.00-8.00)	0.446
HSS			
Pre-op score	58.5 (52.8-64.9)	56.75 (52.5-61.8)	0.070
After 3 months score	90.8 (87.3-92.5)	87.8 (84.890.4)	0.032
After 6 months score	104.12 (67.62-113.25)	104.07 (75-112)	0.969
After 12 months score	110.47 (91.25-113.25)	110.08 (94-112)	0.463
OKS			
Pre-op score	13.50 (7-25)	12.97 (5-21)	0.225
After 3 months score	38.28 (26-44)	38.57 (28-44)	0.465
After 6 months score	42.20 (23-48)	42.80 (26-44)	0.117
After 12 months score	43.83 (38-44)	43.82 (39-44)	1

**Figure 2:**
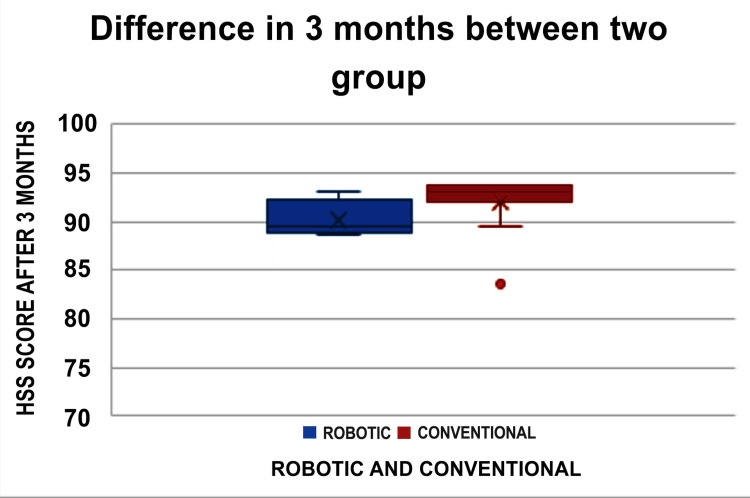
The graph representing the HSS score at three months between the two groups HSS: Hospital for Special Surgery Knee Rating Scale

A comparison of the changes from baseline to postoperative findings between the two groups is shown in Figure 2.

The HSS score at three months and the OKS score at six months showed significant differences, with p-values of 0.004 and 0.037, respectively. However, no significant differences were found in the WOMAC scores from baseline to three, six, or 12 months between the two groups. Similarly, there were no significant differences in the HSS scores from baseline to six or 12 months between the groups. Additionally, no significant differences were observed in the OKS scores from baseline to three or 12 months (Table 3).

**Table 3:** Comparison of changes from baseline to post findings between two groups TKA: total knee arthroplasty; pre-op: pre-operative; WOMAC: Western Ontario McMaster University Osteoarthritis Index; HSS: Hospital for Special Surgery Knee Rating Scale; OKS: Oxford Knee Score

Changes from baseline to post-findings	Group		p-value
	Robotic TKA (n=120)	Conventional TKA (n=120)	
WOMAC			
Pre-op to after 3 months score	60.78 (13-74)	61.44 (31-78)	0.622
Pre-op to after 6 months score	67.48 (42-79)	67.73 (44-83)	0.815
Pre-op to after 12 months score	70.23 (44-81)	69.58 (49-83)	0.464
HSS			
Pre-op to after 3 months score	31.31 (18-54)	34.40 (19.25-60.80)	0.004
Pre-op to after 6 months score	45.96 (17.88-71)	48.10 (24.75-74.50)	<0.125
Pre-op to after 12 months score	52.31 (27-73)	54.11 (34.25-74.50)	0.080
OKS			
Pre-op to after 3 months score	24.78 (13-33)	25.60 (17-37)	0.162
Pre-op to after 6 months score	28.70 (12-37)	29.83 (16-39)	0.037
Pre-op to after 12 months score	30.32 (19-37)	30.86 (22-39)	0.225

## Discussion

Robotic technology improves the accuracy of implant placement by using a surgeon-controlled robotic arm that incorporates preoperative planning, visual feedback, and tactile input, thereby limiting bone resection to predefined stereotactic boundaries [16]. While total hip arthroplasty has a reported failure rate of 5%, TKA shows a higher failure rate of 20% [17]. The most common cause of implant failure is errors in surgeon-controlled positioning. Robotic-assisted knee arthroplasty may lower revision rates by improving implant alignment accuracy, reducing the risk of outliers, and enhancing functional outcomes [18]. Batailler et al. emphasized that the goal of robotic systems is to enhance the precision and reliability of ligament balancing and bone resections, complementing, not replacing, expertly performed surgery [19]. Kafelov et al. suggested that customized alignment and implant positioning can improve functional outcomes in patients undergoing RTKA [20].

Patient satisfaction and reported outcomes for TKA still hold significant potential for improvement. To reduce the likelihood of human error in ligament balancing and component alignment, RTKA was developed [21]. Furthermore, robotic arm-assisted surgery may promote faster functional recovery and shorter hospital stays for TKA patients [22]. Research shows that improved alignment accuracy in RTKA may positively impact radiological results and correlate with superior short-term clinical outcomes [23].

In a comparative study of 20 consecutive RTKAs, Marchand et al. [24] observed a significantly lower mean pain score at six months in the robotic group (p < 0.05). However, this difference in pain scores was not significant after one year. Another postoperative comparative study showed that RTKA led to reduced postoperative pain, faster inflammation reduction, lower analgesic use during hospitalization, and shorter hospital stays compared to conventional jig-based TKA (p < 0.001) [25].

Despite evidence that robotic assistance improves the radiological alignment of components, it remains unclear whether it will positively influence long-term functional outcomes [16]. Our study found no significant difference in postoperative functional satisfaction between patients who underwent RTKA and those who had traditional jig-based TKA at the one-year follow-up. The WOMAC, HSS, and OKS scores at three and 12 months post-surgery showed no significant differences between the two groups. However, when comparing changes from baseline to postoperative results, the HSS score at three months and the OKS score at six months revealed significant differences, with p-values of 0.004 and 0.037, respectively. Our study demonstrated significant improvements in the WOMAC, HSS, and OKS scores when compared to baseline values following both robotic and conventional TKA procedures.

A limitation of our study is the comparison of outcomes between two different TKA implants within each group. Despite this, the implants used in jig-based procedures have shown comparable results over the past decade at our hospital. Additionally, this study was conducted with a one-year follow-up, providing an early clinical evaluation of robotic surgery in comparison to jig-based surgery, grounded in the traditional mechanical alignment principle.

## Conclusions

This study demonstrated that RTKA provided significant improvements in early functional outcomes, as evidenced by the higher HSS score at three months and the greater improvement in OKS at six months compared to baseline. However, by 12 months, no significant differences were observed between RTKA and conventional jig-based TKA in terms of functional outcomes and pain relief. These findings suggest that while robotic technology may enhance early recovery, its long-term benefits remain uncertain. Further research with extended follow-up periods especially in personalized alignment philosophies is necessary to evaluate the potential advantages of robotic assistance in long-term functional outcomes and implant longevity.
